# Supplementary material for: Hot spot prediction in protein-protein interactions by an ensemble system
Source: BMC Syst Biol. 2018 Dec 31;12(Suppl 9):132. doi: 10.1186/s12918-018-0665-8 (PMC6311905; doi:10.1186/s12918-018-0665-8)
Supplement: Supplementary file 2 — All relevant MATLAB code. A simple Matlab implement of our predictor. (ZIP 19,742 kb) [file 12918_2018_665_MOESM2_ESM.zip › Additional file 2/Read ASA/ASA website/SKEMPI/1A22B.htm]

 NetSurfP - Protein Surface Accessibility and Secondary Structure Predictions

  

|  |  |  |
| --- | --- | --- |
|  |  | NetSurfP - Protein Surface Accessibility and Secondary Structure Predictions  Technical University of Denmark |

  


---

```
# For publication of results, please cite:
# A generic method for assignment of reliability scores applied to solvent accessibility predictions.
# Bent Petersen, Thomas Nordahl Petersen, Pernille Andersen, Morten Nielsen and Claus Lundegaard
# BMC Structural Biology 2009, 9:51 doi:10.1186/1472-6807-9-51
#
# Column 1: Class assignment - B for buried or E for Exposed - Threshold: 25% exposure, but not based on RSA
# Column 2: Amino acid
# Column 3: Sequence name
# Column 4: Amino acid number
# Column 5: Relative Surface Accessibility - RSA
# Column 6: Absolute Surface Accessibility
# Column 7: Z-fit score for RSA prediction
# Column 8: Probability for Alpha-Helix
# Column 9: Probability for Beta-strand
# Column 10: Probability for Coil
E P  1A22B                  1    0.554  78.613  -1.298   0.003   0.003   0.994
E K  1A22B                  2    0.612 125.868   0.343   0.004   0.197   0.799
E F  1A22B                  3    0.437  87.706   0.143   0.004   0.420   0.576
B T  1A22B                  4    0.087  12.053  -0.074   0.004   0.616   0.381
E K  1A22B                  5    0.355  72.962   0.842   0.001   0.900   0.099
B C  1A22B                  6    0.041   5.827  -0.988   0.001   0.959   0.040
B R  1A22B                  7    0.232  53.059   1.387   0.000   0.983   0.017
B S  1A22B                  8    0.121  14.228  -0.635   0.001   0.959   0.040
E P  1A22B                  9    0.373  52.872   0.486   0.004   0.616   0.381
E E  1A22B                 10    0.539  94.198  -0.410   0.004   0.085   0.910
E R  1A22B                 11    0.346  79.326   0.662   0.004   0.085   0.910
E E  1A22B                 12    0.424  73.985  -0.202   0.004   0.514   0.481
B T  1A22B                 13    0.255  35.382   0.615   0.002   0.816   0.182
B F  1A22B                 14    0.024   4.757   0.885   0.001   0.900   0.099
B S  1A22B                 15    0.222  25.995   1.194   0.001   0.959   0.040
B C  1A22B                 16    0.019   2.597   1.273   0.001   0.959   0.040
B H  1A22B                 17    0.174  31.687   0.798   0.006   0.962   0.032
B W  1A22B                 18    0.052  12.434  -0.005   0.001   0.900   0.099
E T  1A22B                 19    0.480  66.631   1.329   0.003   0.718   0.279
E L  1A22B                 20    0.421  77.048  -0.107   0.004   0.197   0.799
E G  1A22B                 21    0.472  37.107  -1.016   0.005   0.015   0.979
E P  1A22B                 22    0.665  94.378  -0.374   0.005   0.015   0.979
E I  1A22B                 23    0.385  71.280  -0.810   0.004   0.085   0.910
E Q  1A22B                 24    0.577 103.106  -1.077   0.004   0.138   0.858
B L  1A22B                 25    0.184  33.727  -0.818   0.005   0.336   0.660
E F  1A22B                 26    0.322  64.565   0.173   0.004   0.616   0.381
B Y  1A22B                 27    0.083  17.844   0.569   0.002   0.816   0.182
B T  1A22B                 28    0.138  19.210   0.009   0.001   0.900   0.099
B R  1A22B                 29    0.051  11.725   0.297   0.001   0.959   0.040
B R  1A22B                 30    0.140  32.174   0.267   0.001   0.959   0.040
B N  1A22B                 31    0.101  14.786  -0.119   0.001   0.959   0.040
B T  1A22B                 32    0.202  27.990   0.345   0.001   0.959   0.040
E Q  1A22B                 33    0.317  56.563  -0.479   0.001   0.900   0.099
E E  1A22B                 34    0.365  63.713  -0.214   0.022   0.552   0.426
E W  1A22B                 35    0.512 123.016  -1.259   0.004   0.138   0.858
E T  1A22B                 36    0.574  79.614  -1.722   0.018   0.019   0.964
E Q  1A22B                 37    0.702 125.306  -0.416   0.018   0.047   0.935
E E  1A22B                 38    0.559  97.570   0.630   0.021   0.279   0.699
E W  1A22B                 39    0.471 113.275  -0.391   0.021   0.451   0.528
E K  1A22B                 40    0.397  81.745   1.067   0.022   0.552   0.426
E E  1A22B                 41    0.508  88.713   0.946   0.021   0.451   0.528
B C  1A22B                 42    0.069   9.730  -0.346   0.004   0.197   0.799
E P  1A22B                 43    0.466  66.182  -0.750   0.004   0.138   0.858
E D  1A22B                 44    0.537  77.353  -0.755   0.021   0.279   0.699
B Y  1A22B                 45    0.279  59.665  -0.804   0.022   0.359   0.619
E V  1A22B                 46    0.319  48.969  -0.992   0.004   0.420   0.576
E S  1A22B                 47    0.582  68.269  -1.108   0.005   0.336   0.660
E A  1A22B                 48    0.618  68.126  -2.389   0.004   0.138   0.858
E G  1A22B                 49    0.446  35.061  -0.772   0.004   0.138   0.858
E E  1A22B                 50    0.564  98.583  -0.229   0.004   0.420   0.576
B N  1A22B                 51    0.216  31.578  -0.649   0.004   0.616   0.381
E S  1A22B                 52    0.270  31.597   0.372   0.002   0.816   0.182
B C  1A22B                 53    0.034   4.802  -1.176   0.001   0.900   0.099
E Y  1A22B                 54    0.275  58.703   0.778   0.001   0.900   0.099
B F  1A22B                 55    0.039   7.787   0.026   0.001   0.900   0.099
E N  1A22B                 56    0.259  37.947   0.228   0.004   0.616   0.381
B S  1A22B                 57    0.268  31.363  -0.706   0.021   0.279   0.699
E S  1A22B                 58    0.611  71.586  -1.715   0.018   0.088   0.893
E F  1A22B                 59    0.540 108.418  -1.211   0.004   0.085   0.910
B T  1A22B                 60    0.127  17.615  -1.748   0.004   0.138   0.858
E S  1A22B                 61    0.435  51.005  -1.000   0.004   0.197   0.799
E I  1A22B                 62    0.416  77.052  -1.153   0.005   0.262   0.733
E W  1A22B                 63    0.386  92.713  -1.245   0.004   0.420   0.576
B I  1A22B                 64    0.244  45.159  -0.038   0.003   0.718   0.279
E P  1A22B                 65    0.398  56.490   0.581   0.001   0.900   0.099
B Y  1A22B                 66    0.039   8.292   0.315   0.001   0.959   0.040
B C  1A22B                 67    0.176  24.696   1.379   0.001   0.959   0.040
B I  1A22B                 68    0.018   3.312   1.394   0.001   0.959   0.040
B K  1A22B                 69    0.114  23.409   1.331   0.001   0.959   0.040
B L  1A22B                 70    0.017   3.149   1.355   0.001   0.959   0.040
E T  1A22B                 71    0.246  34.051   1.471   0.001   0.959   0.040
B S  1A22B                 72    0.034   4.008  -1.488   0.001   0.959   0.040
E N  1A22B                 73    0.374  54.724   0.118   0.003   0.718   0.279
E G  1A22B                 74    0.300  23.610  -1.162   0.005   0.262   0.733
E G  1A22B                 75    0.504  39.673  -1.343   0.004   0.138   0.858
E T  1A22B                 76    0.532  73.719  -1.687   0.004   0.197   0.799
E V  1A22B                 77    0.380  58.345  -1.577   0.004   0.197   0.799
E D  1A22B                 78    0.569  81.964  -0.600   0.004   0.197   0.799
E E  1A22B                 79    0.529  92.434  -1.429   0.004   0.420   0.576
E K  1A22B                 80    0.395  81.334   0.121   0.003   0.718   0.279
E C  1A22B                 81    0.425  59.670  -0.443   0.002   0.816   0.182
B F  1A22B                 82    0.085  17.100   0.673   0.002   0.816   0.182
E S  1A22B                 83    0.438  51.287   1.140   0.003   0.718   0.279
B V  1A22B                 84    0.126  19.382  -0.539   0.023   0.655   0.322
E D  1A22B                 85    0.379  54.571  -0.231   0.022   0.552   0.426
E E  1A22B                 86    0.462  80.624  -0.598   0.022   0.552   0.426
B I  1A22B                 87    0.261  48.211   0.429   0.023   0.655   0.322
B V  1A22B                 88    0.096  14.724  -0.361   0.023   0.655   0.322
E Q  1A22B                 89    0.339  60.617   0.916   0.021   0.451   0.528
B P  1A22B                 90    0.164  23.314  -0.920   0.004   0.138   0.858
E D  1A22B                 91    0.496  71.531  -0.797   0.005   0.015   0.979
B P  1A22B                 92    0.125  17.766  -0.401   0.003   0.003   0.994
B P  1A22B                 93    0.118  16.730  -2.178   0.005   0.015   0.979
E I  1A22B                 94    0.480  88.855  -0.569   0.004   0.085   0.910
E A  1A22B                 95    0.470  51.794  -0.639   0.005   0.262   0.733
B L  1A22B                 96    0.123  22.595   1.178   0.004   0.514   0.481
E N  1A22B                 97    0.414  60.639   0.315   0.002   0.816   0.182
B W  1A22B                 98    0.097  23.353   0.330   0.001   0.900   0.099
E T  1A22B                 99    0.420  58.240   0.834   0.001   0.900   0.099
E L  1A22B                100    0.351  64.286  -0.060   0.001   0.900   0.099
B L  1A22B                101    0.251  46.013  -0.302   0.002   0.816   0.182
E N  1A22B                102    0.598  87.533  -1.218   0.005   0.336   0.660
E G  1A22B                103    0.608  47.865  -2.071   0.004   0.085   0.910
E I  1A22B                104    0.528  97.698  -0.838   0.004   0.138   0.858
E H  1A22B                105    0.497  90.459  -0.987   0.004   0.085   0.910
E A  1A22B                106    0.354  38.967  -0.419   0.004   0.138   0.858
B D  1A22B                107    0.259  37.307   0.398   0.003   0.718   0.279
B I  1A22B                108    0.028   5.124  -0.188   0.001   0.900   0.099
B Q  1A22B                109    0.143  25.468   0.975   0.001   0.959   0.040
B V  1A22B                110    0.018   2.736   1.169   0.001   0.959   0.040
B R  1A22B                111    0.117  26.747   0.369   0.001   0.900   0.099
B W  1A22B                112    0.035   8.393  -0.637   0.001   0.900   0.099
E E  1A22B                113    0.390  68.116   0.564   0.003   0.718   0.279
E A  1A22B                114    0.346  38.173  -0.077   0.004   0.197   0.799
B P  1A22B                115    0.161  22.846  -0.509   0.004   0.085   0.910
E R  1A22B                116    0.568 130.026  -2.309   0.004   0.085   0.910
E N  1A22B                117    0.394  57.755  -1.653   0.018   0.088   0.893
E A  1A22B                118    0.401  44.212  -1.944   0.018   0.088   0.893
E D  1A22B                119    0.610  87.829  -2.352   0.018   0.047   0.935
E I  1A22B                120    0.331  61.309  -1.498   0.018   0.047   0.935
E Q  1A22B                121    0.599 107.017  -1.938   0.018   0.047   0.935
E K  1A22B                122    0.520 106.882  -1.837   0.004   0.085   0.910
B G  1A22B                123    0.265  20.855  -1.539   0.004   0.197   0.799
B W  1A22B                124    0.193  46.513  -0.810   0.004   0.514   0.481
B M  1A22B                125    0.121  24.152   0.096   0.002   0.816   0.182
B V  1A22B                126    0.172  26.498   0.191   0.001   0.900   0.099
B L  1A22B                127    0.036   6.665  -0.353   0.001   0.959   0.040
E E  1A22B                128    0.219  38.259   1.009   0.001   0.959   0.040
B Y  1A22B                129    0.030   6.432   0.625   0.001   0.959   0.040
B E  1A22B                130    0.156  27.218   1.028   0.001   0.959   0.040
B L  1A22B                131    0.020   3.699   1.201   0.001   0.959   0.040
B Q  1A22B                132    0.220  39.238   1.522   0.001   0.959   0.040
B Y  1A22B                133    0.104  22.310   1.136   0.001   0.959   0.040
E K  1A22B                134    0.279  57.411   1.256   0.001   0.959   0.040
E E  1A22B                135    0.529  92.469   0.890   0.002   0.816   0.182
E V  1A22B                136    0.588  90.314   0.420   0.004   0.138   0.858
E N  1A22B                137    0.738 107.970  -0.077   0.005   0.015   0.979
E E  1A22B                138    0.661 115.442   0.181   0.005   0.015   0.979
E T  1A22B                139    0.692  96.050   0.274   0.018   0.047   0.935
E K  1A22B                140    0.587 120.828   1.339   0.004   0.420   0.576
B W  1A22B                141    0.173  41.558   0.049   0.003   0.718   0.279
E K  1A22B                142    0.423  86.949   0.611   0.001   0.900   0.099
E M  1A22B                143    0.518 103.712  -0.122   0.001   0.900   0.099
E M  1A22B                144    0.314  62.871   0.433   0.001   0.900   0.099
E D  1A22B                145    0.474  68.303  -0.985   0.002   0.816   0.182
E P  1A22B                146    0.436  61.911  -0.963   0.004   0.420   0.576
E I  1A22B                147    0.406  75.110  -1.338   0.005   0.262   0.733
E L  1A22B                148    0.641 117.385  -0.579   0.004   0.138   0.858
E T  1A22B                149    0.490  68.005  -0.552   0.004   0.197   0.799
E T  1A22B                150    0.271  37.532  -0.126   0.004   0.514   0.481
E S  1A22B                151    0.416  48.779   0.653   0.001   0.900   0.099
B V  1A22B                152    0.087  13.357   0.702   0.001   0.959   0.040
E P  1A22B                153    0.290  41.165   0.861   0.001   0.959   0.040
B V  1A22B                154    0.037   5.610  -0.449   0.002   0.816   0.182
E Y  1A22B                155    0.426  91.143   0.231   0.004   0.514   0.481
E S  1A22B                156    0.428  50.173  -0.412   0.004   0.197   0.799
B L  1A22B                157    0.079  14.465  -0.302   0.005   0.045   0.951
E K  1A22B                158    0.513 105.524   1.073   0.004   0.085   0.910
E V  1A22B                159    0.515  79.125  -0.147   0.005   0.015   0.979
E D  1A22B                160    0.652  93.996  -1.264   0.005   0.015   0.979
E K  1A22B                161    0.253  52.124   0.408   0.004   0.197   0.799
E E  1A22B                162    0.457  79.768   1.702   0.002   0.816   0.182
B Y  1A22B                163    0.069  14.767   1.006   0.001   0.959   0.040
B E  1A22B                164    0.207  36.163   2.016   0.001   0.959   0.040
B V  1A22B                165    0.016   2.475   1.257   0.001   0.959   0.040
B R  1A22B                166    0.097  22.167   1.297   0.001   0.959   0.040
B V  1A22B                167    0.015   2.229   1.457   0.001   0.959   0.040
B R  1A22B                168    0.091  20.793   0.735   0.001   0.959   0.040
B S  1A22B                169    0.016   1.852   0.789   0.001   0.900   0.099
B K  1A22B                170    0.164  33.653   1.373   0.003   0.718   0.279
B Q  1A22B                171    0.210  37.560  -0.196   0.004   0.420   0.576
E R  1A22B                172    0.506 115.966  -0.559   0.018   0.047   0.935
E N  1A22B                173    0.734 107.399  -1.782   0.003   0.003   0.994
E S  1A22B                174    0.614  71.996  -1.742   0.005   0.015   0.979
E G  1A22B                175    0.423  33.251  -1.251   0.005   0.015   0.979
E N  1A22B                176    0.436  63.757   0.418   0.018   0.088   0.893
B Y  1A22B                177    0.175  37.462  -0.669   0.018   0.088   0.893
B G  1A22B                178    0.121   9.531  -1.683   0.018   0.047   0.935
E E  1A22B                179    0.579 101.099  -0.264   0.018   0.047   0.935
B F  1A22B                180    0.222  44.616  -0.618   0.018   0.047   0.935
B S  1A22B                181    0.155  18.107  -0.578   0.018   0.047   0.935
E E  1A22B                182    0.471  82.214  -0.310   0.004   0.138   0.858
E V  1A22B                183    0.375  57.607  -1.021   0.004   0.420   0.576
B L  1A22B                184    0.186  34.093   0.902   0.002   0.816   0.182
E Y  1A22B                185    0.389  83.129   0.228   0.001   0.900   0.099
B V  1A22B                186    0.120  18.398   0.429   0.001   0.900   0.099
E T  1A22B                187    0.448  62.096   0.813   0.001   0.900   0.099
B L  1A22B                188    0.192  35.174   0.697   0.004   0.420   0.576
E P  1A22B                189    0.319  45.224  -0.736   0.004   0.138   0.858
E Q  1A22B                190    0.566 101.034  -0.558   0.004   0.197   0.799
E M  1A22B                191    0.311  62.151  -1.448   0.020   0.205   0.775
E S  1A22B                192    0.781  91.568  -1.484   0.003   0.003   0.994

Explain the output.  Go back.
```

---
